# Association of SGLT2 inhibitors with post-ablation atrial fibrillation recurrence in individuals with heart failure or type 2 diabetes mellitus: a systematic review and meta-analysis

**DOI:** 10.3389/fcvm.2025.1710123

**Published:** 2025-11-21

**Authors:** Jiaqi Li, Fan Zhang, Shaobo Fan, Yingchuan Yan, Jie Zhang, Enyuan Zhang, Dongyan Wu, Fengmin Lu, Jing Xu, Wei Ma

**Affiliations:** Chest Hospital, Tianjin University, Tianjin, China

**Keywords:** sodium–glucose cotransporter-2 inhibitor, atrial fibrillation, catheter ablation, recurrence, type 2 diabetes mellitus, heart failure

## Abstract

**Background:**

The impact of sodium–glucose cotransporter-2 inhibitors (SGLT2i) on post-ablation atrial fibrillation (AF) recurrence is still unclear. Accordingly, we investigated whether exposure to SGLT2i reduces post-ablation AF recurrence among individuals with heart failure (HF) or type 2 diabetes mellitus (T2DM).

**Methods:**

We carried out a structured search of PubMed, Embase, and the Cochrane Library from database launch through August 17, 2025. Pooled analyses were generated with RevMan 5.4 and Stata 18.

**Results:**

11 studies were included, comprising 2 randomized controlled trials (RCTs) and 9 retrospective cohort studies, with a total of 7,664 individuals. Among them, 3,390 received SGLT2i therapy, and 4,274 received non-SGLT2i therapy. Compared with the non-SGLT2i, SGLT2i was linked to decreased post-ablation AF recurrence (RR: 0.61, 95% CI: 0.52–0.71, *p* < 0.001). Subgroup analyses showed consistent reductions in recurrence risk in individuals with AF and T2DM (RR: 0.74, 95% CI: 0.68–0.80, *p* < 0.001) as well as those with AF and HF (RR: 0.61, 95% CI: 0.50–0.74, *p* < 0.001). Furthermore, SGLT2i corresponded to reduced all-cause mortality (RR: 0.66, 95% CI: 0.48–0.91, *p* = 0.010), fewer rehospitalization (RR: 0.79, 95% CI: 0.72–0.88, *p* < 0.001), and a lower incidence of thromboembolic events (RR: 0.56, 95% CI: 0.36–0.86, *p* = 0.009).

**Conclusions:**

Use of SGLT2i was linked to reduced post-ablation AF recurrence, and this association was consistent in AF individuals with T2DM as well as those with HF. Additionally, SGLT2i therapy correlated with reduced risks of all-cause mortality, rehospitalization, and thromboembolic events.

**Systematic Review Registration:**

identifier CRD420251125971.

## 1 Introduction

Atrial fibrillation (AF) populations often present concurrent type 2 diabetes mellitus (T2DM) and heart failure (HF), a combination linked to diminished quality of life together with elevated risks of hospitalization and all-cause mortality (1, 2). Accumulating evidence indicates that, in AF, rhythm-control strategies conferred greater clinical benefit than rate control. Accordingly, for symptomatic individuals who remain refractory to drug therapy, catheter ablation is now widely regarded as the treatment of choice (3). Although catheter ablation techniques have advanced, post-ablation AF recurrence remains widespread and presents a persistent clinical challenge.

Sodium–glucose cotransporter 2 inhibitors (SGLT2i) were initially introduced as antihyperglycaemic agents for the treatment of T2DM. Beyond glycemic control, multiple large randomized trials and meta-analyses have shown that SGLT2i lower heart failure hospitalizations and cardiovascular mortality, independent of T2DM status at study entry (4). This evidence has been recognized in the European Society of Cardiology HF guidelines, which recommend SGLT2i as one of the four foundational therapies for HF (5). The evidence points to a potential cardioprotective role of SGLT2i. Notably, across recent studies, use of SGLT2i has correlated with fewer new-onset AF events in individuals with HF or T2DM (6). Despite these advances, evidence regarding SGLT2i and post-ablation AF recurrence remains limited.

Therefore, our objective was to determine if SGLT2i therapy reduces post-ablation AF recurrence among individuals with HF or T2DM and to evaluate its impact on other cardiovascular endpoints following ablation.

## 2 Methods

The review was prepared in accordance with PRISMA, and the prespecified protocol was prospectively registered on PROSPERO (CRD420251125971).

### 2.1 Inclusion and exclusion criteria

Inclusion (PICOS). Population: individuals with AF who underwent catheter ablation; Intervention: Treatment with SGLT2i; Comparator: non-SGLT2i therapy; Outcomes: primary—post-ablation AF recurrence; secondary—all-cause mortality, hospitalization, and thromboembolic events. Eligible studies had to provide the primary outcome; Study design: Randomized controlled trials (RCTs) or observational studies. Exclusion. Irrelevant studies, reviews, case reports, conference abstracts, etc.; Studies including AF individuals receiving SGLT2i therapy but not undergoing catheter ablation; Studies lacking access to original data or full-text articles.

### 2.2 Search strategy

Guided by predefined inclusion/exclusion criteria, electronic searches spanned PubMed, Embase, and the Cochrane Library from their launch to 17 August 2025. The search strategy used three concept blocks combined with Boolean operators: (Atrial Fibrillation OR Auricular Fibrillation) AND (Sodium–Glucose Transporter 2 Inhibitor OR Sodium–Glucose Cotransporter 2 Inhibitor OR SGLT-2 Inhibitor OR Canagliflozin OR Dapagliflozin OR Empagliflozin OR Ipragliflozin OR Luseogliflozin OR Ertugliflozin OR Sotagliflozin) AND (Catheter Ablation OR Ablation). Moreover, we hand-checked the references of all eligible studies to ensure completeness of the retrieval.

### 2.3 Study selection and data extraction

Database searches were performed independently by two reviewers (ZF and FAB). After deduplication, we examined titles and abstracts to ascertain potentially eligible studies, then appraised the full texts of records deemed relevant. Eligibility determinations followed prespecified inclusion/exclusion criteria, and disagreements were settled by a third reviewer (YYC). From the eligible studies, we extracted the following data items: (1) first author, year of publication, study design, sample sizes of the SGLT2i and non-SGLT2i groups, mean age, sex distribution, percentage with persistent AF, ablation strategy, and follow-up duration; (2) clinical outcomes, including AF recurrence rate, all-cause mortality, rehospitalization, and thromboembolic events.

### 2.4 Quality assessment

For RCTs, risk of bias was assessed with the Cochrane tool, covering random sequence generation, allocation concealment, blinding of participants/personnel and outcome assessors, incomplete outcome data, selective reporting, and other sources of bias; each domain was graded as low, high, or unclear risk. We appraised the quality of observational studies using the Newcastle–Ottawa Scale (NOS), with a score of seven or higher considered indicative of high-quality research. It should be noted that these tools were employed solely to assess methodological rigor and did not serve as criteria for study inclusion or exclusion**.**

### 2.5 Statistical analysis

As all outcomes of interest were dichotomous variables, effect estimates were reported as relative risks (RRs) with 95% confidence intervals (CIs). Heterogeneity across studies was examined using Cochran's *Q* and *I*^2^ statistic. A random-effects model was employed under substantial heterogeneity (*p* < 0.10 for *Q* or *I*^2^ > 50%); when these conditions did not hold, a fixed-effect model was employed. Subgrouping was defined by the presence of T2DM or HF among patients with AF. Sensitivity analysis was performed when notable heterogeneity was present. For any outcomes synthesized from ten or more studies, potential publication bias was examined via funnel-plot inspection and Egger's test (*α* = 0.05). Statistical significance was defined as *p* < 0.05. Data synthesis and statistical computations were executed in RevMan 5.4 and Stata 18.

## 3 Results

### 3.1 Search results

**Figure 1:**
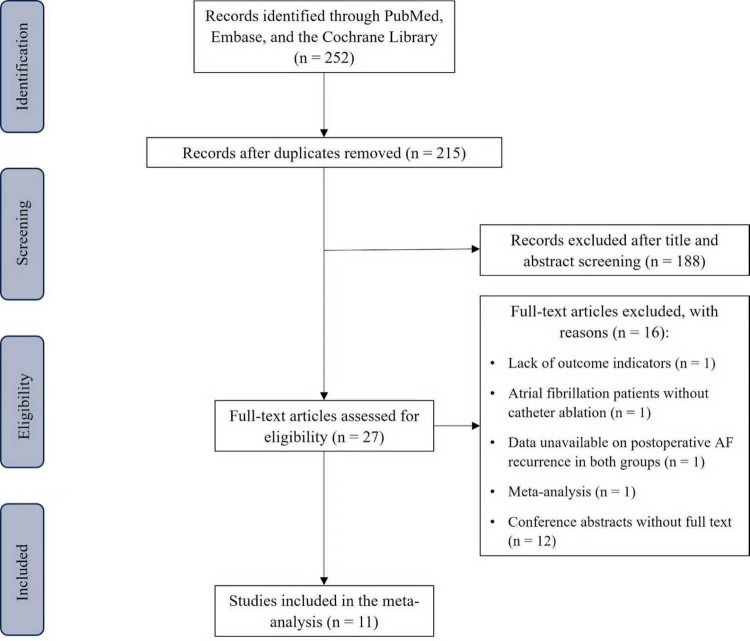
Flow diagram of literature screening and results.

Overall, 252 citations were retrieved. Deduplication reduced the set to 215; 188 failed title/abstract screening, and 27 received full-text assessment. Following detailed assessment, 11 studies met the criteria and were included in the synthesis, comprising 2 RCTs (7, 8), and 9 retrospective cohort studies (9–17). Altogether, 7,664 individuals were analysed, including 3,390 who received SGLT2i therapy and 4,274 who received non-SGLT2i therapy. Figure 1 summarizes the study identification and screening process.

### 3.2 Baseline characteristics

Although Zhao et al. published two studies in 2023 (16) and 2025 (9) using data derived from the Chinese Atrial Fibrillation Registry, they covered distinct time periods and study populations, we analyzed them as separate comparisons within the meta-analysis. Moreover, outcomes were evaluated with propensity score–matched cohorts in Abu-Qaoud et al. (17) and in two studies by Zhao et al. (9, 16). In the studies conducted by Zhao et al. (9), Okajima et al. (10), Harada et al. (7), and Cetin et al. (12), the target population consisted of individuals with AF and HF. In contrast, the studies by Qi et al. (13), Luo et al. (15), Zhao et al. (16), Abu-Qaoud et al. (17), and Kishima et al. (8) focused on AF individuals with T2DM. Both Harada et al. (7) and Qi et al. (13) restricted their analyses to individuals with persistent AF. Most of the included trials utilized both cryoballoon and radiofrequency energy sources for ablation. For paroxysmal AF, the predominant ablation strategy was bilateral pulmonary vein antral isolation. For persistent AF, beyond the above treatment, additional linear ablation was performed as indicated. Individuals with concomitant atrial flutter also underwent cavotricuspid isthmus ablation. A summary of baseline characteristics for each study is shown in Table 1.

**Table 1:** Baseline characteristics of the included studies.

Study	Year	Study type	Country	Intervention	Sample size, *n*	Age (years, mean ± SD)	Male, *n* (%)	PeAF, *n* (%)	Ablation strategy	T2DM prevalence in HF, *n* (%)	HF prevalence in T2DM, *n* (%)	Follow-up (months)
Zhao et al. (9)	2025	Retrospective cohort	China	SGLT2i	368	63.5 ± 9.8	242 (65.8)	279 (75.8)	For all patients: CPVI; For PeAF: additional linear ablations; For AFL: CTI ablation	182 (49.5)	NA	27.5
				non-SGLT2i	368	62.7 ± 10.9	239 (64.9)	291 (79.1)		198 (53.8)		
Okajima et al. (10)	2025	Retrospective cohort	Japan	SGLT2i	45	NA	NA	NA	For all patients: CPVI; Additional ablation was performed when necessary	21 (15.0)	NA	12.4
				non-SGLT2i	96	NA	NA	NA				
Harada et al. (7)	2025	RCT	Japan	SGLT2i	51	70.2 ± 8.3	33 (64.8)	51 (100.0)	For the first session of PeAF ablation: CPVI; For AFL: CTI ablation	0 (0.0)	NA	12.0
				non-SGLT2i	51	72.0 ± 8.8	38 (74.5)	51 (100.0)		0 (0.0)		
Hakgor et al. (11)	2025	Retrospective cohort	Turkey	SGLT2i	211	59.8 ± 9.9	129 (61.1)	87 (41.2)	For all patients: CPVI + CTI ablation; Additional linear ablations, complex fractionated atrial electrograms, and antrum of the superior vena cava at the operator's discretion	NA	NA	24.0
				non-SGLT2i	403	57.1 ± 9.7	286 (56.1)	112 (27.8)				
Cetin et al. (12)	2025	Retrospective cohort	Turkey	SGLT2i	71	65.1 ± 7.9	43 (60.6)	37 (52.1)	For all patients: CPVI	26 (36.6)	NA	11.5
				non-SGLT2i	175	63.1 ± 10.0	109 (62.3)	97 (55.4)		57 (32.6)		
Qi et al. (13)	2024	Retrospective cohort	China	SGLT2i	91	70.9 ± 0.7	52 (57.0)	91 (100.0)	For all patients: CPVI; Rescue ablation was performed as necessary	NA	3 (3.0)	16.2
				non-SGLT2i	91	67.3 ± 0.9	48 (53.0)	91 (100.0)			5 (5.0)	
Noh et al. (14)	2024	Retrospective cohort	South Korea	dapagliflozin	73	73.5 ± 4.8	61 (83.6)	30 (41.1)	For all patients: CPVI; Additional linear ablations, and antrum of the superior vena cava at the operator's discretion	NA	NA	18.0
				control	199	71.7 ± 5.6	175 (87.9)	92 (46.2)				
Luo et al. (15)	2024	Retrospective cohort	China	dapagliflozin	79	63.4 ± 10.4	48 (60.8)	35 (44.3)	For all patients: CPVI; For PeAF: additional linear ablation; For AFL: CTI ablation	NA	NA	15.5
				control	247	63.8 ± 9.9	144 (58.3)	119 (48.2)				
Zhao et al. (16)	2023	Retrospective cohort	China	SGLT2i	138	63.9 ± 8.7	98 (71.0)	64 (46.4)	For all patients: CPVI; For PeAF: dditional linear ablation;	NA	29 (21.0)	15.5
				non-SGLT2i	387	64.0 ± 9.5	276 (71.3)	203 (49.6)			57 (14.0)	
Abu-Qaoud et al. (17)	2023	Retrospective cohort	United States	SGLT2i	2,225	65.0 ± 9.0	1648 (75.0)	NA	For all patients: CPVI; Additional linear ablations for remaining after completion of pulmonary vein isolation	NA	1,316 (59)	12.0
				non-SGLT2i	2,225	65.0 ± 9.0	1642 (74.0)	NA			1,302 (58)	
Kishima et al. (8)	2022	RCT	Japan	Tofogliflozin	38	70.3 ± 8.6	26 (68.0)	22 (58.0)	For all patients: CPVI; Additional superior vena cava isolation or CTI ablation were conducted at the operator's discretion	NA	10 (26.0)	12.0
				Anagliptin	32	70.3 ± 7.7	22 (69.0)	18 (56.0)			9 (28.0)	

T2DM, type 2 diabetes mellitus; HF, heart failure; SGLT2i, sodium–glucose cotransporter 2 inhibitors; CPVI, circumferential pulmonary vein isolation; PeAF, persistent atrial fibrillation; AFL, atrial flutter; CTI, cavotricuspid isthmus; RCT, randomized controlled trials.

### 3.3 Primary outcomes

#### 3.3.1 AF recurrence

**Figure 2:**
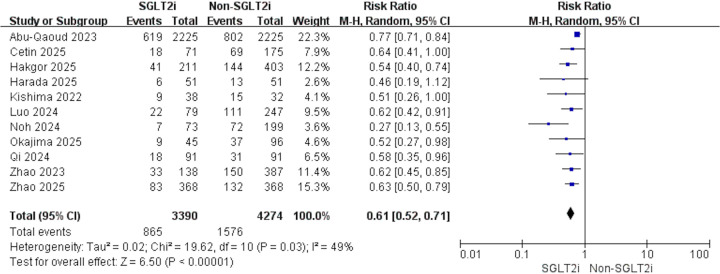
Forest plot illustrating the impact of SGLT2i vs. non-SGLT2i on AF recurrence following catheter ablation.

11 studies contributed data: 3,390 treated with SGLT2i vs. 4,274 comparators with non-SGLT2i. Heterogeneity analysis indicated moderate variability among studies (*p* = 0.03, *I*^2^ = 49%); therefore, a random-effects model was applied. Relative to non-SGLT2i, use of SGLT2i was linked to a reduced risk of post-ablation AF recurrence (RR 0.61, 95% CI 0.52–0.71, *p* < 0.001, Figure 2). Sensitivity analysis confirmed the robustness of this finding (Supplementary Figure S1). When the study by Abu-Qaoud et al. was excluded, *I*^2^ = 0%, and the summary of the remaining studies showed no reversal of the result (RR: 0.58, 95% CI: 0.51–0.66, *P* < 0.001). Meta-regression analysis indicated that study design, average age, proportion of males, proportion of persistent AF, average follow-up time, or study population had no effect on the above result (Table 2).

**Table 2:** Meta regression results for AF recurrence.

Study characteristics	Coefficient	95% CI	*P*
Study Design	−0.179	−0.824 to 0.466	0.546
Average Age	−0.282	−0.827 to 0.263	0.260
Male, *n* (%)	0.086	−0.061 to 0.233	0.213
PeAF, *n* (%)	−0.042	−0.770 to 0.687	0.896
Average Follow-up Time	−0.165	−0.347 to 0.017	0.071
Study Population	−0.133	−0.384 to 0.117	0.249

AF, atrial fibrillation; PeAF, persistent atrial fibrillation.

#### 3.3.2 Subgroup analyses

**Figure 3:**
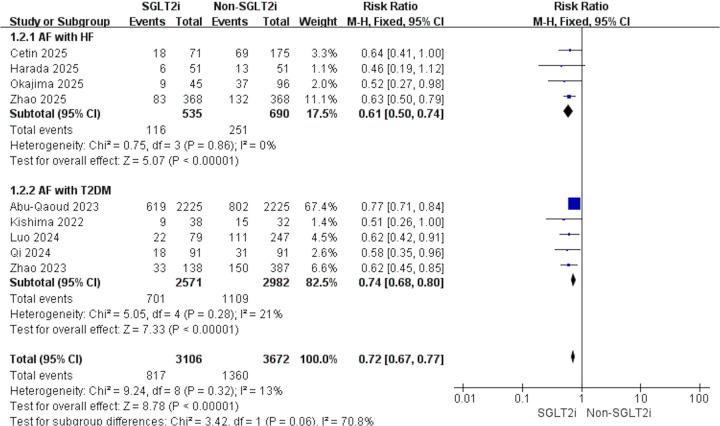
Forest plot of subgroup analyses according to AF with HF or AF with T2DM.

According to the study population, the analysis was divided into AF with HF and AF with T2DM. For AF with HF, 4 studies contributed data (SGLT2i, *n* = 535; non-SGLT2i, *n* = 690). Using a fixed-effects model, SGLT2i were associated with fewer post-ablation AF recurrence (RR: 0.61; 95% CI: 0.50–0.74; *p* < 0.001; *I*^2^ = 0%; Figure 3). For AF with T2DM, another 4 studies (SGLT2i, *n* = 2,571; non-SGLT2i, *n* = 2,982) yielded concordant findings, indicating a decreased risk of recurrence with SGLT2i (RR: 0.74; 95% CI: 0.68–0.80; *p* < 0.001; *I*^2^ = 21%; Figure 3). Subgroup analysis results based on study design, average age, proportion of males, proportion of persistent AF, and average follow-up time are shown in Table 3.

**Table 3:** Results of subgroup analysis for AF recurrence.

Study characteristics	Number of studies	RR (95% CI)	*I* ^2^	*P*
Study Design				
RCT	2	0.49 (0.28, 0.84)	0%	0.009
Retrospective cohort	9	0.61 (0.52, 0.72)	55%	<0.001
Average Age				
≥70	3	0.39 (0.26, 0.61)	0%	<0.001
<70	6	0.66 (0.58, 0.76)	43%	<0.001
Male, *n* (%)				
≥70	4	0.57 (0.39, 0.83)	73%	0.003
<70	6	0.60 (0.52, 0.69)	0%	<0.001
PeAF, *n* (%)				
≥50	5	0.61 (0.51, 0.73)	0%	<0.001
<50	4	0.55 (0.43, 0.70)	38%	<0.001
Follow-up (months)				
≥15	6	0.58 (0.50, 0.67)	8%	<0.001
<15	5	0.71 (0.60, 0.83)	14%	<0.001

AF, atrial fibrillation; PeAF, persistent atrial fibrillation.

### 3.4 Secondary outcomes

#### 3.4.1 All-cause mortality

**Figure 4:**
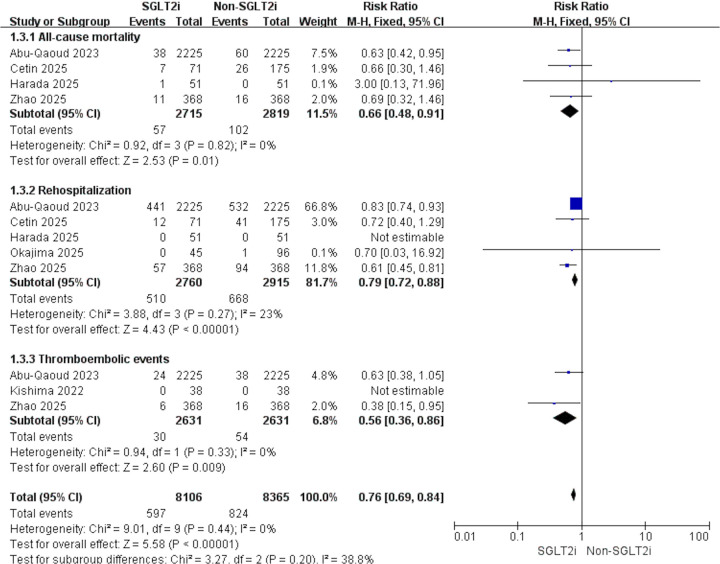
Forest plot illustrating the impact of SGLT2i vs. non-SGLT2i on all-cause mortality, rehospitalization, and thromboembolic events after AF catheter ablation.

4 studies were eligible for this outcome. With no evidence of heterogeneity (*p* = 0.82, *I*^2^ = 0%), pooled results demonstrated that SGLT2i significantly reduced all-cause mortality after AF ablation (RR: 0.66, 95% CI: 0.49–0.91, *p* = 0.01, Figure 4).

#### 3.4.2 Rehospitalization

5 studies contributed data to this outcome. Given the low heterogeneity across studies (*p* = 0.27, *I*^2^ = 23%), the results indicated that SGLT2i was linked to a decreased risk of rehospitalization compared with non-SGLT2i (RR: 0.79, 95% CI: 0.72–0.88, *p* < 0.001, Figure 4).

#### 3.4.3 Thromboembolic events

3 studies assessed this outcome. Heterogeneity testing showed no evidence of variability among studies (*p* = 0.33, *I*^2^ = 0%). Consistently, individuals receiving SGLT2i experienced fewer thromboembolic events after catheter ablation (RR: 0.56, 95% CI: 0.36–0.86, *p* = 0.009, Figure 4).

### 3.5 Quality assessment and publication bias

The Cochrane risk-of-bias assessment identified a high risk for random sequence generation in Harada et al. (7) (Supplementary Table S1). All retrospective cohorts achieved NOS scores ≥7, indicating high methodological quality (Supplementary Table S2).

The funnel plot for AF recurrence appeared asymmetric on visual inspection (Supplementary Figure S2). Egger's test (*t* = −3.92, *P* < 0.05) and Harbord's regression (*t* = −3.63, *P* < 0.05) was significant, suggesting potential publication bias. To account for this, we implemented the trim-and-fill method under a random-effects model, which yielded log RR: −0.266 (95% CI: −0.329 to −0.203). Adjusted analysis suggested that publication bias did not meaningfully alter the overall results (Supplementary Figure S3).

## 4 Discussion

This meta-analysis demonstrated that, relative to non-SGLT2 regimens, the incidence of post-ablation AF recurrence was lower among individuals receiving SGLT2i. Unlike prior meta-analyses that mainly investigated individuals with AF and T2DM, this study was the first to synthesize evidence from AF individuals with concomitant HF in subgroup analyses, thereby confirming that the protective effect extends to this group as well. In addition, SGLT2i was linked to reduced risks of all-cause mortality, rehospitalization, and thromboembolic events in this population.

The earliest randomized trial on this question, by Kishima et al. (8) in 2022, found fewer post-ablation AF recurrences with tofogliflozin vs. anagliptin in AF individuals with T2DM. Shortly thereafter, Using the TriNetX network, Abu-Qaoud et al. (17) conducted a large retrospective cohort analysis that corroborated these findings. More recently, a meta-analysis pooling 6 studies further reinforced the evidence that SGLT2i decreased the likelihood of post-ablation AF recurrence in this population (18). Consistent with these observations, the present analysis incorporated additional recent studies and reached similar conclusions. However, this conclusion applied only to individuals with AF with T2DM. SGLT2i have broad pharmacological actions, and multiple randomized trials have confirmed their ability to reduce HF hospitalization and cardiovascular mortality (4). Given the well-recognized bidirectional relationship between AF and HF, investigators have increasingly examined whether these benefits extend to AF individuals with concomitant HF. Four studies published in 2025 consistently demonstrated that SGLT2i decreased the risk of recurrence in this subgroup. By synthesizing these findings, our meta-analysis was the first to comprehensively evaluate SGLT2i in AF individuals with HF, thereby underscoring their potential value in lowering recurrence risk after catheter ablation and extending their applicability beyond AF with T2DM.

Evidence from RCTs and meta-analyses indicated a reduction in atrial arrhythmia risk with SGLT2i therapy among individuals with T2DM or HF (6). Several potential pathophysiological mechanisms may account for this effect: Inflammatory signaling and atrial fibrotic remodeling are central contributors to the initiation and maintenance of atrial fibrillation. SGLT2i may mitigate oxidative injury, augment mitochondrial respiration, and normalize intracellular Ca^2+^ homeostasis, thereby facilitating reverse structural and electrophysiological remodeling (19); they have also been shown to reduce epicardial adipose tissue and suppress inflammatory responses (20). Since ionic imbalance is closely related to arrhythmogenesis, SGLT2i may improve potassium balance (21). Specifically, empagliflozin has been reported to attenuate Ca^2+^/calmodulin-dependent protein kinase II signaling and reduce sarcoplasmic-reticulum Ca^2+^ leak, thereby stabilizing cardiomyocyte Ca^2+^ handling (22). In addition, by inhibiting proximal tubular reabsorption of glucose and sodium, SGLT2i exert a diuretic effect that reduces left atrial pressure, which may help prevent AF (7). Beyond these direct mechanisms, SGLT2i also mitigate glycemic variability, facilitate weight reduction, and blunt sympathetic overactivation, all of which contribute to suppression of AF development (23–25).

Nevertheless, the preventive effect of SGLT2i on AF remains controversial. *Post hoc* analysis of DAPA-HF found no relation to a reduced risk of incident AF with dapagliflozin (26). Similarly, a recent meta-analysis concluded that SGLT2i failed to decrease AF incidence in individuals with cardiometabolic disease (27). However, AF events in these studies were not prespecified as primary outcomes, and in some cases were not confirmed by electrocardiographic documentation, which could have introduced misclassification. Moreover, the overall incidence of AF in these cohorts was very low, possibly limiting the ability to detect a true effect of SGLT2i. Notably, even within meta-analysis reporting predominantly negative findings, sensitivity analysis suggested that empagliflozin may exert a protective effect against AF (28).

The cardiovascular benefits of SGLT2i extend beyond their potential role in preventing AF. The study by Mariani et al. (29) included 198 HF patients with reduced ejection fraction who received Implantable Cardioverter Defibrillator or Cardiac Resynchronization Therapy Defibrillator. After using SGLT2i, the incidence of atrial and ventricular arrhythmias was significantly reduced compared to before treatment, with a particularly pronounced reduction in atrial fibrillation and non-sustained ventricular tachycardia in the subgroup analysis. Multicenter observational evidence suggested that SGLT2i correlated with reduced rates of HF hospitalization and all-cause mortality among the AF/T2DM population (2). Furthermore, Patel et al. (30) indicated that SGLT2i use was linked to decreased major adverse cardiovascular events across a broad population, particularly reducing deaths from HF and sudden cardiac death. Although there is still controversy regarding whether SGLT2i can reduce thrombosis formation, a study by Mohamed et al. (31) found that while SGLT2i increased the incidence of erythrocytosis in HF patients, it was not associated with an increased risk of thromboembolic events over one year. Furthermore, a study by Chang et al. (32) suggested that SGLT2i may reduce the thromboembolic risk in individuals with T2DM and AF by alleviating inflammation in AF individuals. Our meta-analysis showed declines in postoperative all-cause mortality, rehospitalization, and thromboembolic events, indicating that these benefits persist in individuals undergoing catheter ablation.

## 5 Limitations

(1) We included only two RCTs, whereas the remainder consisted of retrospective cohorts, rendering them susceptible to selection bias. (2) Although subgroup analyses were performed, only the study by Harada et al. specifically enrolled AF individuals with HF but without T2DM; the other studies included varying proportions of individuals with T2DM or HF, which may have impacted the subgroup analyses. (3) Although ablation strategies varied across studies, the distribution of approaches did not differ significantly between treatment arms. (4) Most of the included studies used radiofrequency and cryoballoon as the energy sources for ablation to varying degrees, and they did not clearly specify the number of individuals treated with each type of energy. Therefore, a subgroup analysis based on ablation energy types could not be performed. Pulsed-field ablation has developed rapidly due to its advantages, including speed, high tissue selectivity, and fewer complications. However, there are currently no studies on the use of SGLT2i after AF pulsed-field ablation. As a result, the impact of SGLT2i on AF recurrence following ablation with different energy types remains unclear. Future studies are needed to update and further validate and supplement the conclusions. (5) Due to the limited data, we could not assess the efficacy of different types of SGLT2i on post-ablation AF recurrence, nor evaluate potential differences in recurrence rates across different HF subtypes. Additionally, no subgroup analyses were performed for secondary outcomes.

## 6 Conclusions

This meta-analysis indicated that use of SGLT2 inhibitors was linked to reduced post-ablation AF recurrence relative to non-SGLT2i regimens. This effect was consistent across AF individuals with T2DM and those with HF. Moreover, SGLT2i was linked to lower risks of all-cause mortality, rehospitalization, and thromboembolic events following ablation.

## Data Availability

The original contributions presented in the study are included in the article/Supplementary Material, further inquiries can be directed to the corresponding author.
