# Supplementary material for: Association of SGLT2 inhibitors with post-ablation atrial fibrillation recurrence in individuals with heart failure or type 2 diabetes mellitus: a systematic review and meta-analysis
Source: Front Cardiovasc Med. 2025 Nov 21;12:1710123. doi: 10.3389/fcvm.2025.1710123 (PMC12678333; doi:10.3389/fcvm.2025.1710123)
Supplement: Supplementary file 1 [file Datasheet1.pdf]

Supplementary Material

1 Supplementary Figures

1.1 Supplementary Figure 1. Sensitivity analysis for AF recurrence.

1.2 Supplementary Figure 2. Funnel plot assessing publication bias for AF recurrence.

1.3 Supplementary Figure 3. Trim-and-fill analysis evaluating the influence of publication bias on AF recurrence.

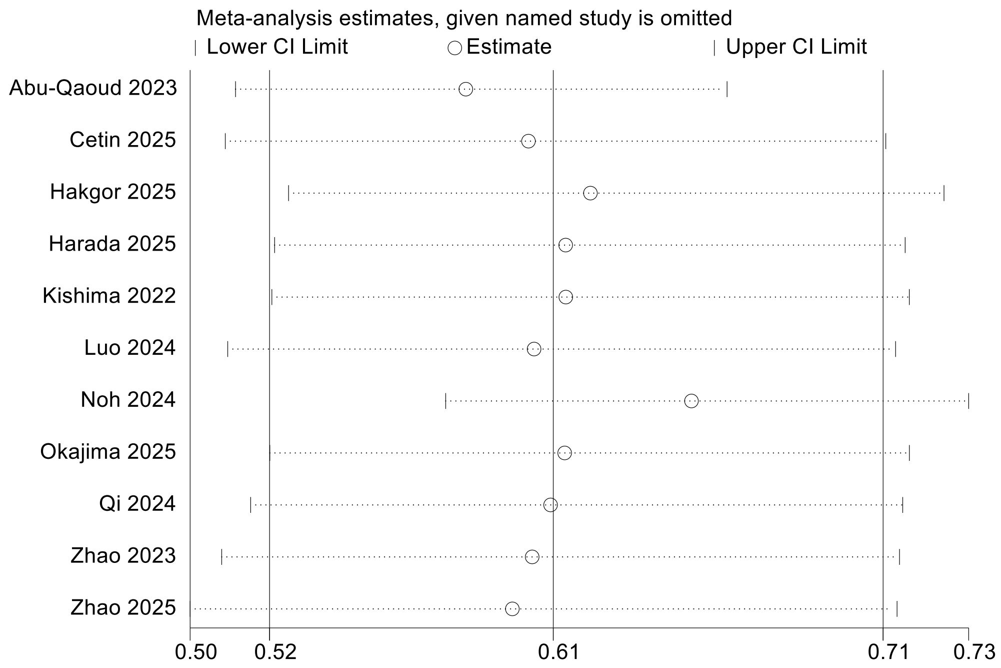

Supplementary Figure 1. Sensitivity analysis for AF recurrence.

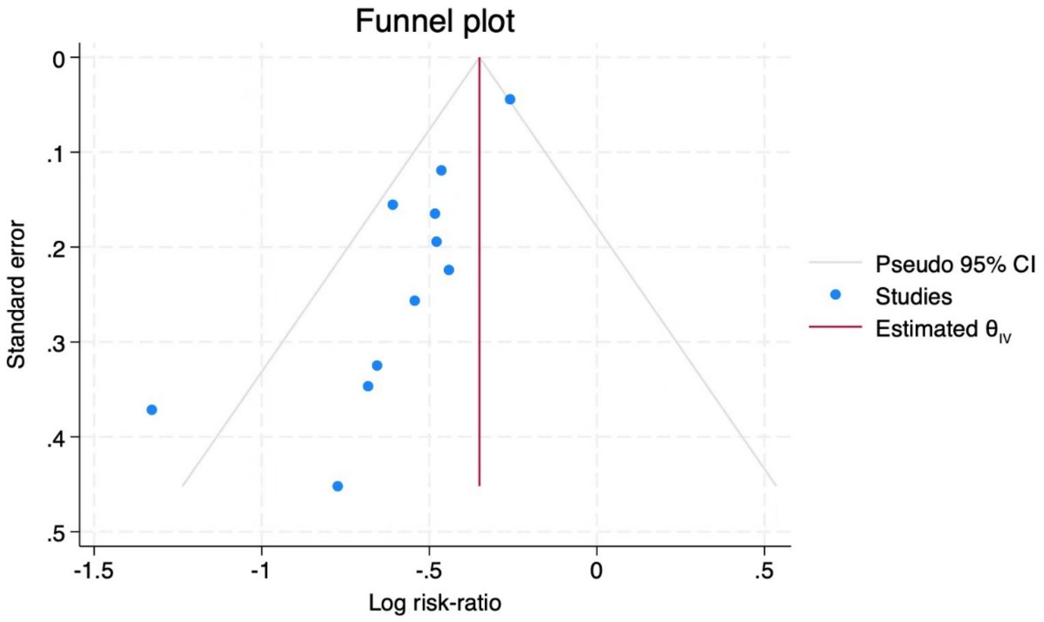

Supplementary Figure 2. Funnel plot assessing publication bias for AF recurrence.

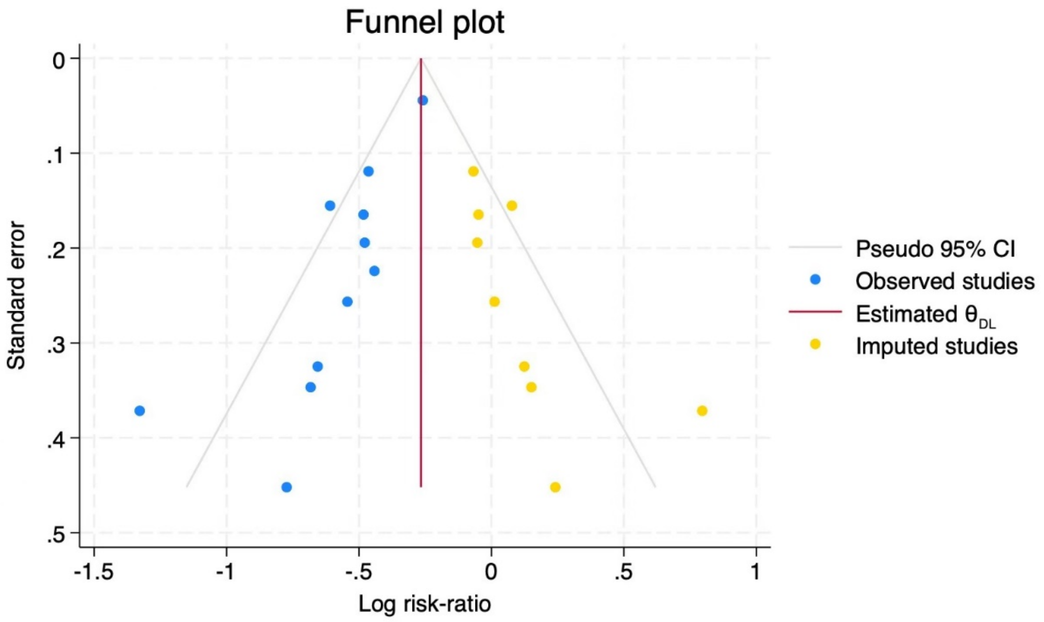

Supplementary Figure 3. Trim-and-fill analysis evaluating the influence of publication bias on AF recurrence.

2 Supplementary Tables

2.1 Supplementary Table 1. Quality assessment of RCTs.

2.2 Supplementary Table 2. Quality assessment of retrospective cohort studies.

Study	Selection bias		Performance bias	Detection bias	Attrition bias	Reporting bias	Other bias
	Random sequence generation	Allocation concealment	Blinding of participants and personnel	Blinding of outcome assessment	Incomplete outcome data	Selective reporting	Other sources of bias
Harada et al. [7]	High risk	Unclear risk	Low risk	Low risk	Low risk	Low risk	Low risk
Kishima et al. [8]	Low risk	Unclear risk	Low risk	Low risk	Low risk	Low risk	Low risk

Supplementary Table 1. Quality assessment of RCTs

RCTs: randomized controlled trials

Supplementary Table 2. Quality assessment of retrospective cohort studies

Study	Selection				Comparability		Outcome			
	Representative-ness	Selection of non-exposed	Ascertainment of exposure	Outcome not Present at start	Comparability on most important tactors	Comparability on other risk factors	Assessment of outcome	Long enough follow-up	Adequacy (completeness) of follow-up	Total score
Zhao et al. [9]	1	1	1	1	1	1	1	1	1	9
Okajima et al. [10]	1	1	1	1	0	0	1	1	1	7
Hakgor et al. [11]	1	1	1	1	0	1	1	1	1	8
Cetin et al. [12]	1	1	1	1	1	1	1	1	1	9
Qi et al. [13]	1	1	1	1	1	1	1	1	1	9
Noh et al. [14]	1	1	1	1	1	1	1	1	1	9
Luo et al. [15]	1	1	1	1	1	1	1	1	1	9
Zhao et al. [16]	1	1	1	1	1	1	1	1	1	9
Abu-Qaoud et al. [17]	1	1	1	1	1	1	1	1	1	9
